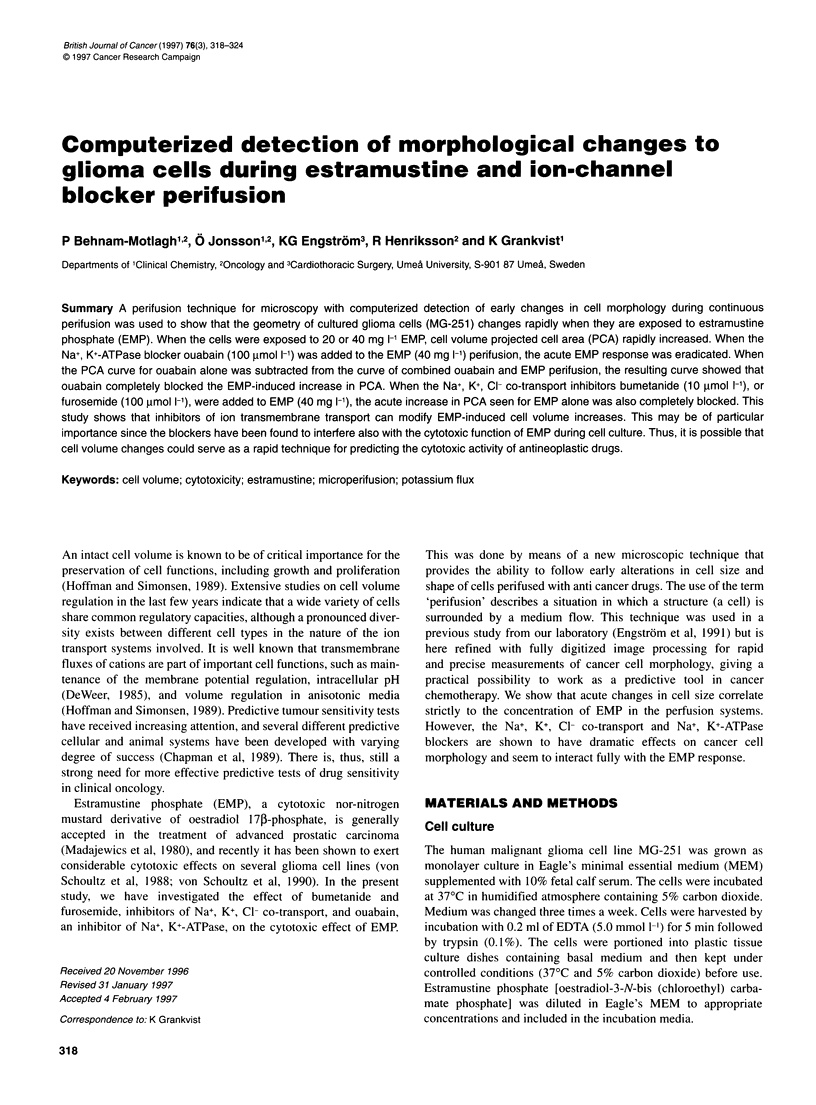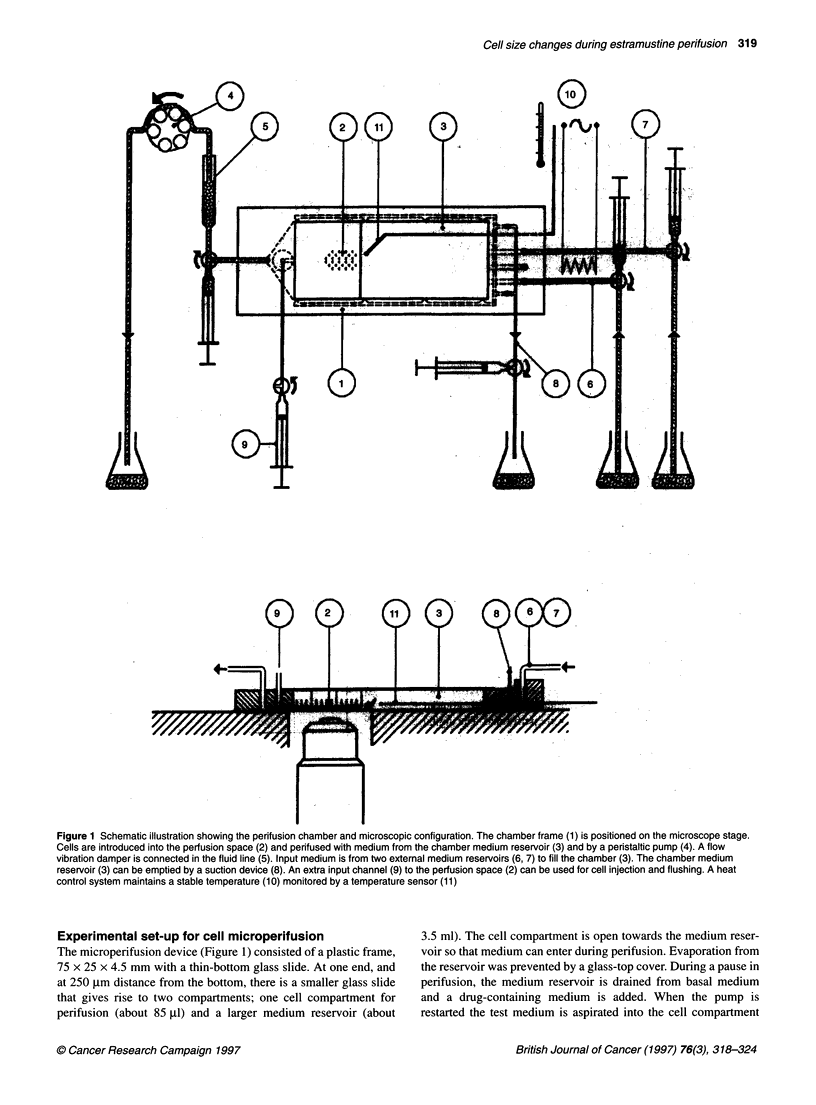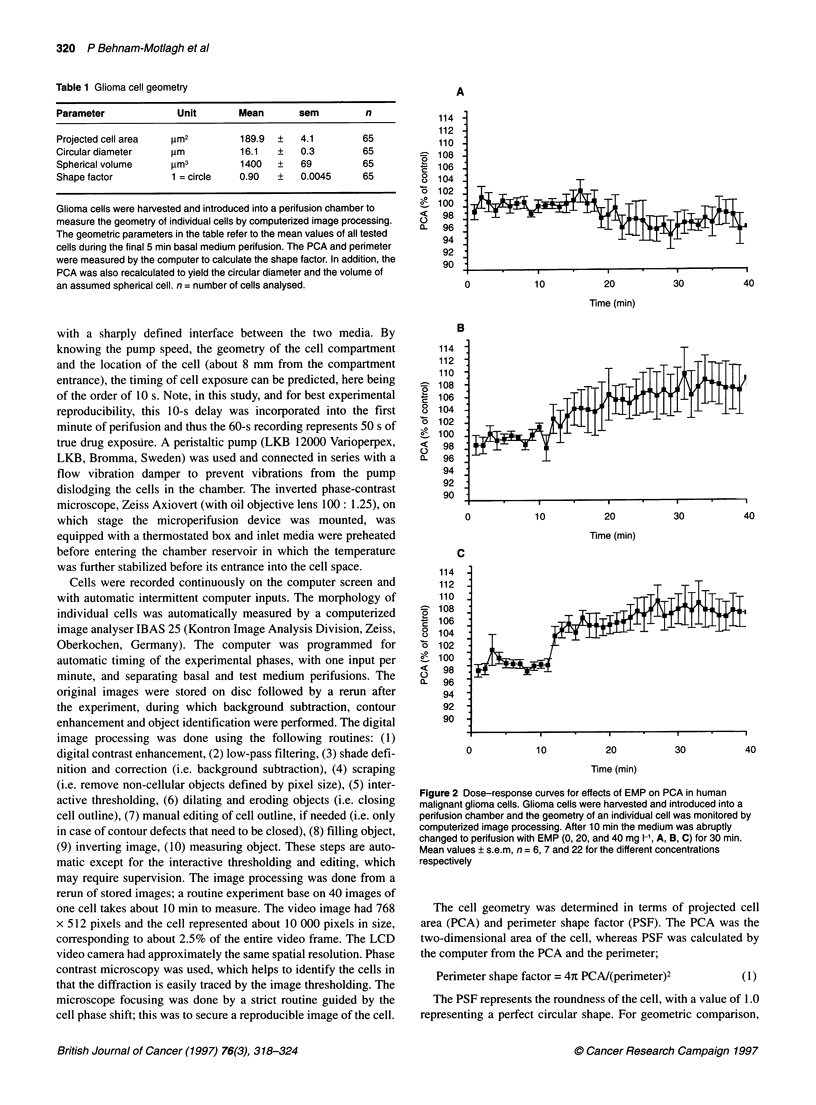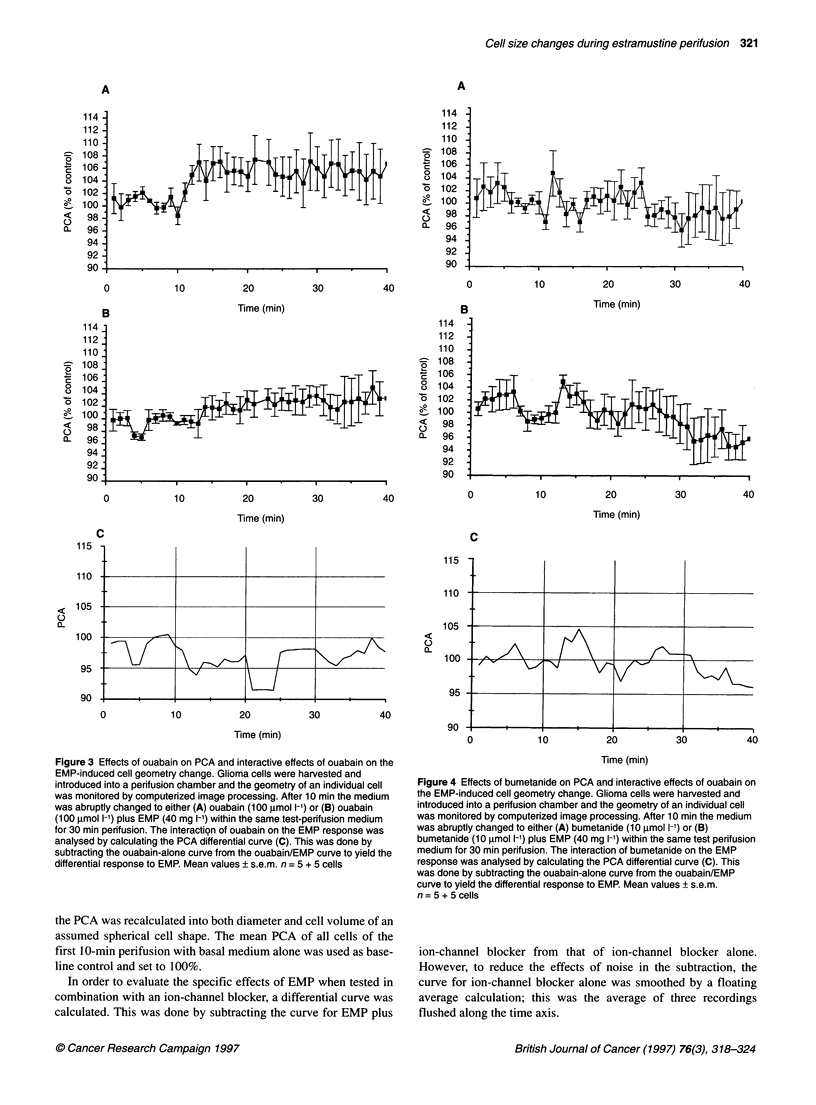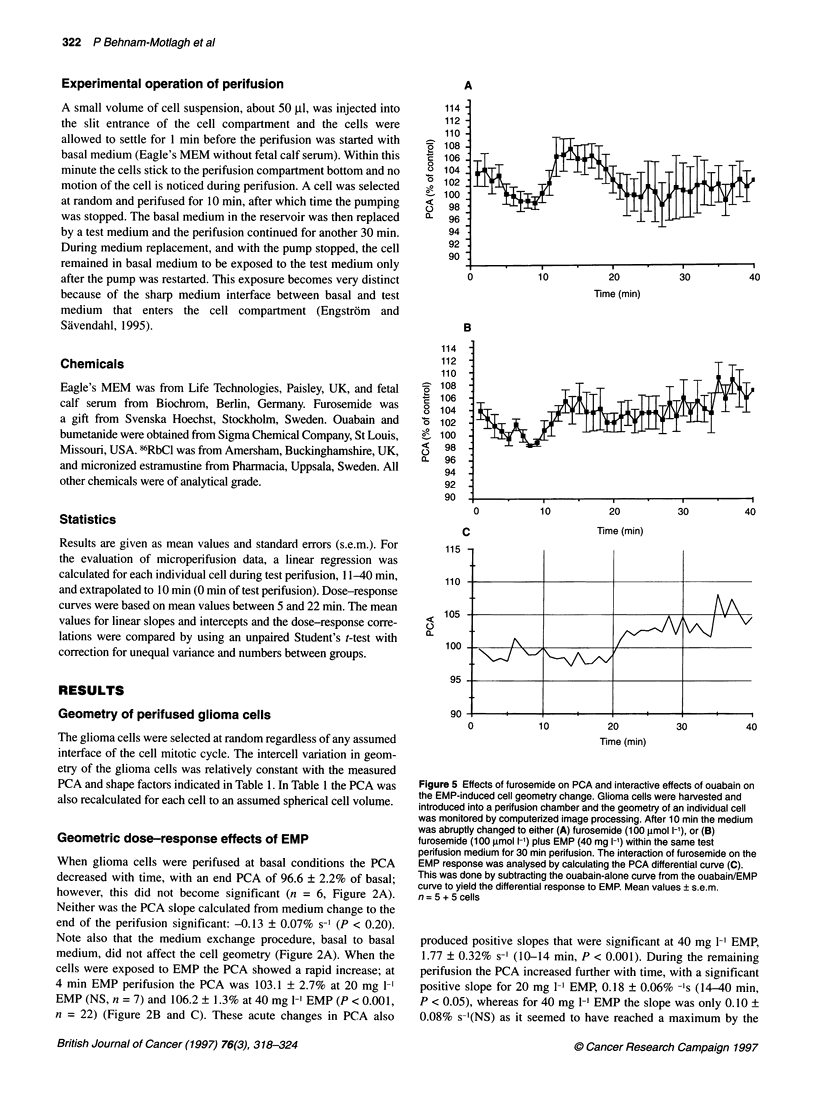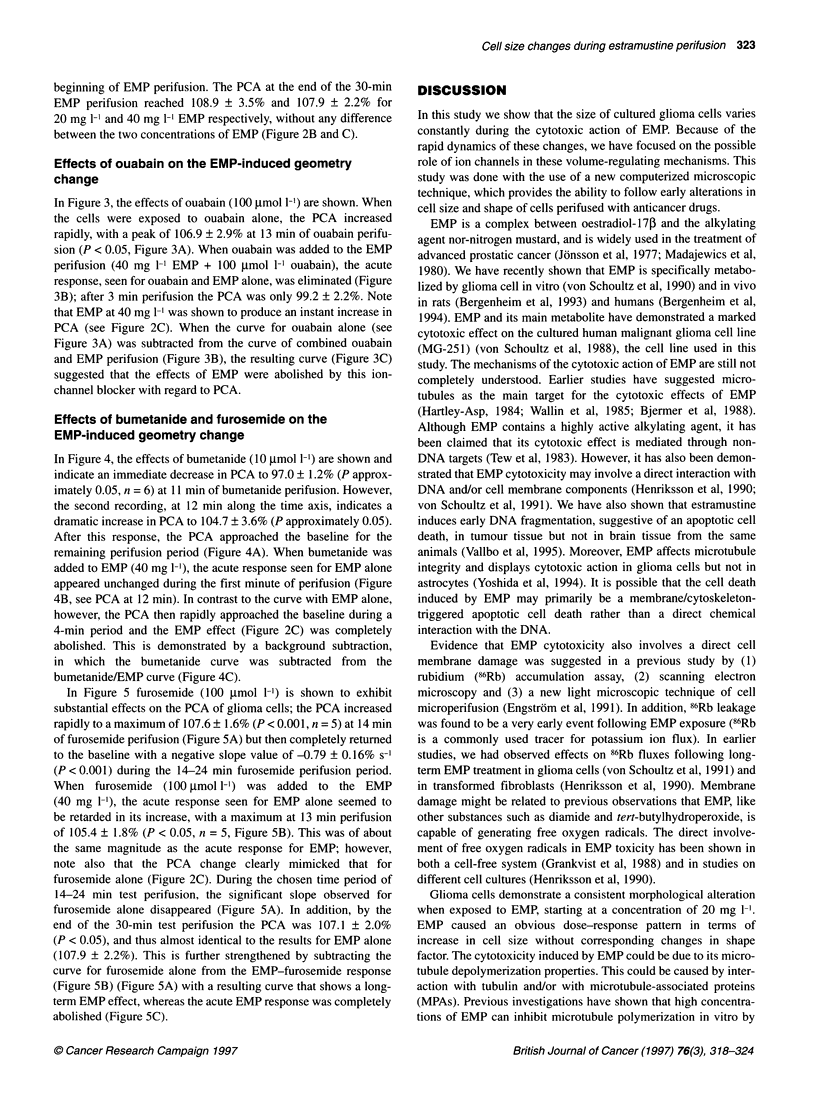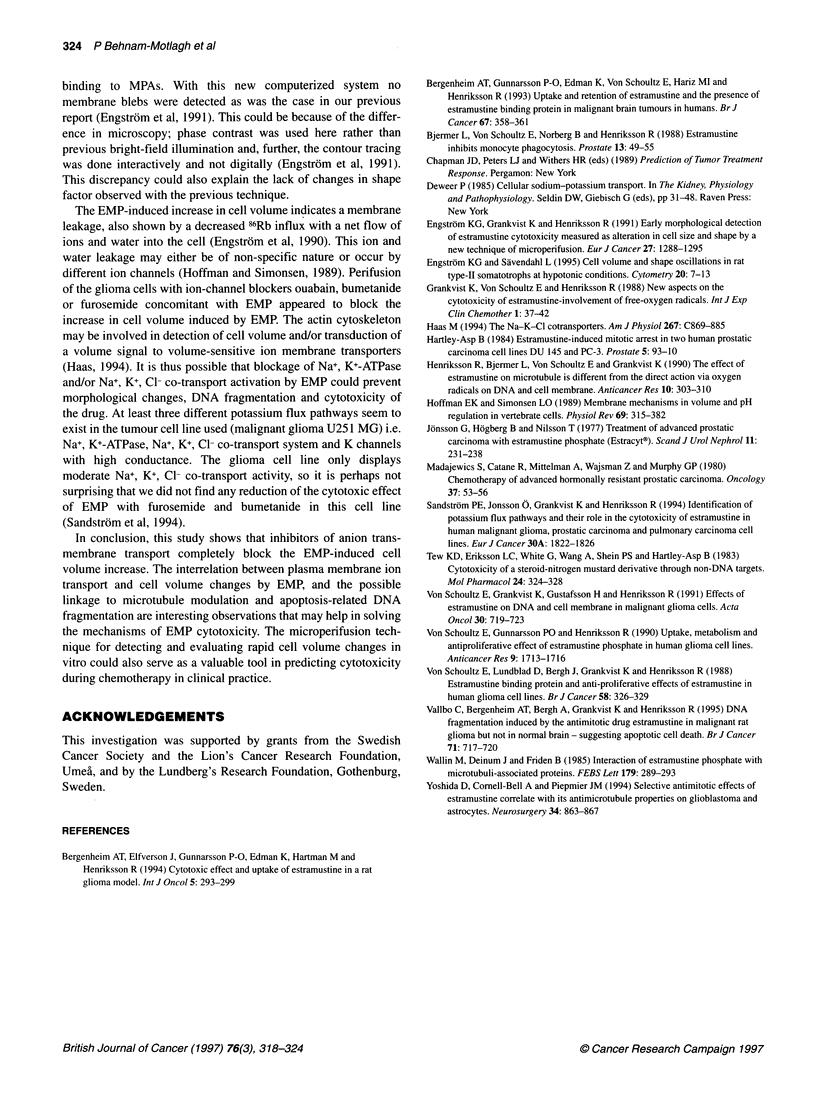# Computerized detection of morphological changes to glioma cells during estramustine and ion-channel blocker perifusion.

**DOI:** 10.1038/bjc.1997.385

**Published:** 1997

**Authors:** P. Behnam-Motlagh, O. Jonsson, K. G. EngstrÃ¶m, R. Henriksson, K. Grankvist

**Affiliations:** Department of Clinical Chemistry, UmeÃ¥ University, Sweden.

## Abstract

A perifusion technique for microscopy with computerized detection of early changes in cell morphology during continuous perifusion was used to show that the geometry of cultured glioma cells (MG-251) changes rapidly when they are exposed to estramustine phosphate (EMP). When the cells were exposed to 20 or 40 mg l(-1) EMP, cell volume projected cell area (PCA) rapidly increased. When the Na+,K+-ATPase blocker ouabain (100 micromol l(-1)) was added to the EMP (40 mg l(-1)) perifusion, the acute EMP response was eradicated. When the PCA curve for ouabain alone was subtracted from the curve of combined ouabain and EMP perifusion, the resulting curve showed that ouabain completely blocked the EMP-induced increase in PCA. When the Na+, K+, Cl- co-transport inhibitors bumetanide (10 micromol l(-1)), or furosemide (100 micromol l(-1)), were added to EMP (40 mg l(-1)), the acute increase in PCA seen for EMP alone was also completely blocked. This study shows that inhibitors of ion transmembrane transport can modify EMP-induced cell volume increases. This may be of particular importance since the blockers have been found to interfere also with the cytotoxic function of EMP during cell culture. Thus, it is possible that cell volume changes could serve as a rapid technique for predicting the cytotoxic activity of antineoplastic drugs.


British Journal of Cancer (1997) **76**(3), 318–324
© 1997 Cancer Research Campaign

### Computerized detection of morphological changes to glioma cells during estramustine and ion-channel blocker perifusion

**P Behnam-Motlagh[1,2], Ö Jonsson[1,2], KG Engström[3], R Henriksson[2] and K Grankvist[1]**

Departments of [1]Clinical Chemistry, [2]Oncology and [3]Cardiothoracic Surgery, Umeå University, S-901 87 Umeå, Sweden

**Summary** A perifusion technique for microscopy with computerized detection of early changes in cell morphology during continuous perifusion was used to show that the geometry of cultured glioma cells (MG-251) changes rapidly when they are exposed to estramustine phosphate (EMP). When the cells were exposed to 20 or 40 mg $l^{-1}$ EMP, cell volume projected cell area (PCA) rapidly increased. When the $Na^+$, $K^+$-ATPase blocker ouabain (100 μmol $l^{-1}$) was added to the EMP (40 mg $l^{-1}$) perifusion, the acute EMP response was eradicated. When the PCA curve for ouabain alone was subtracted from the curve of combined ouabain and EMP perifusion, the resulting curve showed that ouabain completely blocked the EMP-induced increase in PCA. When the $Na^+$, $K^+$, $Cl^-$ co-transport inhibitors bumetanide (10 μmol $l^{-1}$), or furosemide (100 μmol $l^{-1}$), were added to EMP (40 mg $l^{-1}$), the acute increase in PCA seen for EMP alone was also completely blocked. This study shows that inhibitors of ion transmembrane transport can modify EMP-induced cell volume increases. This may be of particular importance since the blockers have been found to interfere also with the cytotoxic function of EMP during cell culture. Thus, it is possible that cell volume changes could serve as a rapid technique for predicting the cytotoxic activity of antineoplastic drugs.

**Keywords:** cell volume; cytotoxicity; estramustine; microperifusion; potassium flux

An intact cell volume is known to be of critical importance for the preservation of cell functions, including growth and proliferation (Hoffman and Simonsen, 1989). Extensive studies on cell volume regulation in the last few years indicate that a wide variety of cells share common regulatory capacities, although a pronounced diversity exists between different cell types in the nature of the ion transport systems involved. It is well known that transmembrane fluxes of cations are part of important cell functions, such as maintenance of the membrane potential regulation, intracellular pH (DeWeer, 1985), and volume regulation in anisotonic media (Hoffman and Simonsen, 1989). Predictive tumour sensitivity tests have received increasing attention, and several different predictive cellular and animal systems have been developed with varying degree of success (Chapman et al, 1989). There is, thus, still a strong need for more effective predictive tests of drug sensitivity in clinical oncology.

Estramustine phosphate (EMP), a cytotoxic nor-nitrogen mustard derivative of oestradiol 17β-phosphate, is generally accepted in the treatment of advanced prostatic carcinoma (Madajewics et al, 1980), and recently it has been shown to exert considerable cytotoxic effects on several glioma cell lines (von Schoultz et al, 1988; von Schoultz et al, 1990). In the present study, we have investigated the effect of bumetanide and furosemide, inhibitors of $Na^+$, $K^+$, $Cl^-$ co-transport, and ouabain, an inhibitor of $Na^+$, $K^+$-ATPase, on the cytotoxic effect of EMP.

*Received 20 November 1996*
*Revised 31 January 1997*
*Accepted 4 February 1997*

*Correspondence to:* K Grankvist

This was done by means of a new microscopic technique that provides the ability to follow early alterations in cell size and shape of cells perifused with anti cancer drugs. The use of the term 'perifusion' describes a situation in which a structure (a cell) is surrounded by a medium flow. This technique was used in a previous study from our laboratory (Engström et al, 1991) but is here refined with fully digitized image processing for rapid and precise measurements of cancer cell morphology, giving a practical possibility to work as a predictive tool in cancer chemotherapy. We show that acute changes in cell size correlate strictly to the concentration of EMP in the perfusion systems. However, the $Na^+$, $K^+$, $Cl^-$ co-transport and $Na^+$, $K^+$-ATPase blockers are shown to have dramatic effects on cancer cell morphology and seem to interact fully with the EMP response.

### MATERIALS AND METHODS

### Cell culture

The human malignant glioma cell line MG-251 was grown as monolayer culture in Eagle's minimal essential medium (MEM) supplemented with 10% fetal calf serum. The cells were incubated at 37°C in humidified atmosphere containing 5% carbon dioxide. Medium was changed three times a week. Cells were harvested by incubation with 0.2 ml of EDTA (5.0 mmol $l^{-1}$) for 5 min followed by trypsin (0.1%). The cells were portioned into plastic tissue culture dishes containing basal medium and then kept under controlled conditions (37°C and 5% carbon dioxide) before use. Estramustine phosphate [oestradiol-3-*N*-bis (chloroethyl) carbamate phosphate] was diluted in Eagle's MEM to appropriate concentrations and included in the incubation media.

**Figure 1** Schematic illustration showing the perifusion chamber and microscopic configuration. The chamber frame (1) is positioned on the microscope stage. Cells are introduced into the perfusion space (2) and perifused with medium from the chamber medium reservoir (3) and by a peristaltic pump (4). A flow vibration damper is connected in the fluid line (5). Input medium is from two external medium reservoirs (6, 7) to fill the chamber (3). The chamber medium reservoir (3) can be emptied by a suction device (8). An extra input channel (9) to the perfusion space (2) can be used for cell injection and flushing. A heat control system maintains a stable temperature (10) monitored by a temperature sensor (11)

### Experimental set-up for cell microperifusion

The microperifusion device (Figure 1) consisted of a plastic frame, 75 × 25 × 4.5 mm with a thin-bottom glass slide. At one end, and at 250 μm distance from the bottom, there is a smaller glass slide that gives rise to two compartments; one cell compartment for perifusion (about 85 μl) and a larger medium reservoir (about 3.5 ml). The cell compartment is open towards the medium reservoir so that medium can enter during perifusion. Evaporation from the reservoir was prevented by a glass-top cover. During a pause in perifusion, the medium reservoir is drained from basal medium and a drug-containing medium is added. When the pump is restarted the test medium is aspirated into the cell compartment

**Table 1** Glioma cell geometry

| Parameter | Unit | Mean | | sem | n |
|---|---|---|---|---|---|
| Projected cell area | $\mu m^2$ | 189.9 | ± | 4.1 | 65 |
| Circular diameter | µm | 16.1 | ± | 0.3 | 65 |
| Spherical volume | $\mu m^3$ | 1400 | ± | 69 | 65 |
| Shape factor | 1 = circle | 0.90 | ± | 0.0045 | 65 |

Glioma cells were harvested and introduced into a perifusion chamber to measure the geometry of individual cells by computerized image processing. The geometric parameters in the table refer to the mean values of all tested cells during the final 5 min basal medium perifusion. The PCA and perimeter were measured by the computer to calculate the shape factor. In addition, the PCA was also recalculated to yield the circular diameter and the volume of an assumed spherical cell. *n* = number of cells analysed.

**Figure 2** Dose–response curves for effects of EMP on PCA in human malignant glioma cells. Glioma cells were harvested and introduced into a perifusion chamber and the geometry of an individual cell was monitored by computerized image processing. After 10 min the medium was abruptly changed to perifusion with EMP (0, 20, and 40 mg $l^{-1}$, **A**, **B**, **C**) for 30 min. Mean values ± s.e.m, *n* = 6, 7 and 22 for the different concentrations respectively with a sharply defined interface between the two media. By knowing the pump speed, the geometry of the cell compartment and the location of the cell (about 8 mm from the compartment entrance), the timing of cell exposure can be predicted, here being of the order of 10 s. Note, in this study, and for best experimental reproducibility, this 10-s delay was incorporated into the first minute of perifusion and thus the 60-s recording represents 50 s of true drug exposure. A peristaltic pump (LKB 12000 Varioperpex, LKB, Bromma, Sweden) was used and connected in series with a flow vibration damper to prevent vibrations from the pump dislodging the cells in the chamber. The inverted phase-contrast microscope, Zeiss Axiovert (with oil objective lens 100 : 1.25), on which stage the microperifusion device was mounted, was equipped with a thermostated box and inlet media were preheated before entering the chamber reservoir in which the temperature was further stabilized before its entrance into the cell space.

Cells were recorded continuously on the computer screen and with automatic intermittent computer inputs. The morphology of individual cells was automatically measured by a computerized image analyser IBAS 25 (Kontron Image Analysis Division, Zeiss, Oberkochen, Germany). The computer was programmed for automatic timing of the experimental phases, with one input per minute, and separating basal and test medium perifusions. The original images were stored on disc followed by a rerun after the experiment, during which background subtraction, contour enhancement and object identification were performed. The digital image processing was done using the following routines: (1) digital contrast enhancement, (2) low-pass filtering, (3) shade definition and correction (i.e. background subtraction), (4) scraping (i.e. remove non-cellular objects defined by pixel size), (5) interactive thresholding, (6) dilating and eroding objects (i.e. closing cell outline), (7) manual editing of cell outline, if needed (i.e. only in case of contour defects that need to be closed), (8) filling object, (9) inverting image, (10) measuring object. These steps are automatic except for the interactive thresholding and editing, which may require supervision. The image processing was done from a rerun of stored images; a routine experiment base on 40 images of one cell takes about 10 min to measure. The video image had 768 × 512 pixels and the cell represented about 10 000 pixels in size, corresponding to about 2.5% of the entire video frame. The LCD video camera had approximately the same spatial resolution. Phase contrast microscopy was used, which helps to identify the cells in that the diffraction is easily traced by the image thresholding. The microscope focusing was done by a strict routine guided by the cell phase shift; this was to secure a reproducible image of the cell.

The cell geometry was determined in terms of projected cell area (PCA) and perimeter shape factor (PSF). The PCA was the two-dimensional area of the cell, whereas PSF was calculated by the computer from the PCA and the perimeter;

$$\text{Perimeter shape factor} = 4\pi \, \text{PCA}/(\text{perimeter})^2 \qquad (1)$$

The PSF represents the roundness of the cell, with a value of 1.0 representing a perfect circular shape. For geometric comparison,

© Cancer Research Campaign 1997

**Figure 3** Effects of ouabain on PCA and interactive effects of ouabain on the EMP-induced cell geometry change. Glioma cells were harvested and introduced into a perifusion chamber and the geometry of an individual cell was monitored by computerized image processing. After 10 min the medium was abruptly changed to either (**A**) ouabain (100 μmol $l^{-1}$) or (**B**) ouabain (100 μmol $l^{-1}$) plus EMP (40 mg $l^{-1}$) within the same test-perifusion medium for 30 min perifusion. The interaction of ouabain on the EMP response was analysed by calculating the PCA differential curve (**C**). This was done by subtracting the ouabain-alone curve from the ouabain/EMP curve to yield the differential response to EMP. Mean values ± s.e.m. $n$ = 5 + 5 cells

**Figure 4** Effects of bumetanide on PCA and interactive effects of ouabain on the EMP-induced cell geometry change. Glioma cells were harvested and introduced into a perifusion chamber and the geometry of an individual cell was monitored by computerized image processing. After 10 min the medium was abruptly changed to either (**A**) bumetanide (10 μmol $l^{-1}$) or (**B**) bumetanide (10 μmol $l^{-1}$) plus EMP (40 mg $l^{-1}$) within the same test perifusion medium for 30 min perifusion. The interaction of bumetanide on the EMP response was analysed by calculating the PCA differential curve (**C**). This was done by subtracting the ouabain-alone curve from the ouabain/EMP curve to yield the differential response to EMP. Mean values ± s.e.m. $n$ = 5 + 5 cells the PCA was recalculated into both diameter and cell volume of an assumed spherical cell shape. The mean PCA of all cells of the first 10-min perifusion with basal medium alone was used as baseline control and set to 100%.

In order to evaluate the specific effects of EMP when tested in combination with an ion-channel blocker, a differential curve was calculated. This was done by subtracting the curve for EMP plus ion-channel blocker from that of ion-channel blocker alone. However, to reduce the effects of noise in the subtraction, the curve for ion-channel blocker alone was smoothed by a floating average calculation; this was the average of three recordings flushed along the time axis.

### Experimental operation of perifusion

A small volume of cell suspension, about 50 µl, was injected into the slit entrance of the cell compartment and the cells were allowed to settle for 1 min before the perifusion was started with basal medium (Eagle's MEM without fetal calf serum). Within this minute the cells stick to the perifusion compartment bottom and no motion of the cell is noticed during perifusion. A cell was selected at random and perifused for 10 min, after which time the pumping was stopped. The basal medium in the reservoir was then replaced by a test medium and the perifusion continued for another 30 min. During medium replacement, and with the pump stopped, the cell remained in basal medium to be exposed to the test medium only after the pump was restarted. This exposure becomes very distinct because of the sharp medium interface between basal and test medium that enters the cell compartment (Engström and Sävendahl, 1995).

### Chemicals

Eagle's MEM was from Life Technologies, Paisley, UK, and fetal calf serum from Biochrom, Berlin, Germany. Furosemide was a gift from Svenska Hoechst, Stockholm, Sweden. Ouabain and bumetanide were obtained from Sigma Chemical Company, St Louis, Missouri, USA. $^{86}$RbCl was from Amersham, Buckinghamshire, UK, and micronized estramustine from Pharmacia, Uppsala, Sweden. All other chemicals were of analytical grade.

### Statistics

Results are given as mean values and standard errors (s.e.m.). For the evaluation of microperifusion data, a linear regression was calculated for each individual cell during test perifusion, 11–40 min, and extrapolated to 10 min (0 min of test perifusion). Dose–response curves were based on mean values between 5 and 22 min. The mean values for linear slopes and intercepts and the dose–response correlations were compared by using an unpaired Student's *t*-test with correction for unequal variance and numbers between groups.

### RESULTS

### Geometry of perifused glioma cells

The glioma cells were selected at random regardless of any assumed interface of the cell mitotic cycle. The intercell variation in geometry of the glioma cells was relatively constant with the measured PCA and shape factors indicated in Table 1. In Table 1 the PCA was also recalculated for each cell to an assumed spherical cell volume.

### Geometric dose–response effects of EMP

When glioma cells were perifused at basal conditions the PCA decreased with time, with an end PCA of 96.6 ± 2.2% of basal; however, this did not become significant ($n$ = 6, Figure 2A). Neither was the PCA slope calculated from medium change to the end of the perifusion significant: –0.13 ± 0.07% $s^{-1}$ ($P < 0.20$). Note also that the medium exchange procedure, basal to basal medium, did not affect the cell geometry (Figure 2A). When the cells were exposed to EMP the PCA showed a rapid increase; at 4 min EMP perifusion the PCA was 103.1 ± 2.7% at 20 mg $l^{-1}$ EMP (NS, $n$ = 7) and 106.2 ± 1.3% at 40 mg $l^{-1}$ EMP ($P < 0.001$, $n$ = 22) (Figure 2B and C). These acute changes in PCA also produced positive slopes that were significant at 40 mg $l^{-1}$ EMP, 1.77 ± 0.32% $s^{-1}$ (10–14 min, $P < 0.001$). During the remaining perifusion the PCA increased further with time, with a significant positive slope for 20 mg $l^{-1}$ EMP, 0.18 ± 0.06% $^{-1}$s (14–40 min, $P < 0.05$), whereas for 40 mg $l^{-1}$ EMP the slope was only 0.10 ± 0.08% $s^{-1}$(NS) as it seemed to have reached a maximum by the

**Figure 5** Effects of furosemide on PCA and interactive effects of ouabain on the EMP-induced cell geometry change. Glioma cells were harvested and introduced into a perifusion chamber and the geometry of an individual cell was monitored by computerized image processing. After 10 min the medium was abruptly changed to either (**A**) furosemide (100 µmol $l^{-1}$), or (**B**) furosemide (100 µmol $l^{-1}$) plus EMP (40 mg $l^{-1}$) within the same test perifusion medium for 30 min perifusion. The interaction of furosemide on the EMP response was analysed by calculating the PCA differential curve (**C**). This was done by subtracting the ouabain-alone curve from the ouabain/EMP curve to yield the differential response to EMP. Mean values ± s.e.m. $n$ = 5 + 5 cells

© Cancer Research Campaign 1997

beginning of EMP perifusion. The PCA at the end of the 30-min EMP perifusion reached 108.9 ± 3.5% and 107.9 ± 2.2% for 20 mg $l^{-1}$ and 40 mg $l^{-1}$ EMP respectively, without any difference between the two concentrations of EMP (Figure 2B and C).

### Effects of ouabain on the EMP-induced geometry change

In Figure 3, the effects of ouabain (100 μmol $l^{-1}$) are shown. When the cells were exposed to ouabain alone, the PCA increased rapidly, with a peak of 106.9 ± 2.9% at 13 min of ouabain perifusion ($P < 0.05$, Figure 3A). When ouabain was added to the EMP perifusion (40 mg $l^{-1}$ EMP + 100 μmol $l^{-1}$ ouabain), the acute response, seen for ouabain and EMP alone, was eliminated (Figure 3B); after 3 min perifusion the PCA was only 99.2 ± 2.2%. Note that EMP at 40 mg $l^{-1}$ was shown to produce an instant increase in PCA (see Figure 2C). When the curve for ouabain alone (see Figure 3A) was subtracted from the curve of combined ouabain and EMP perifusion (Figure 3B), the resulting curve (Figure 3C) suggested that the effects of EMP were abolished by this ion-channel blocker with regard to PCA.

### Effects of bumetanide and furosemide on the EMP-induced geometry change

In Figure 4, the effects of bumetanide (10 μmol $l^{-1}$) are shown and indicate an immediate decrease in PCA to 97.0 ± 1.2% ($P$ approximately 0.05, $n = 6$) at 11 min of bumetanide perifusion. However, the second recording, at 12 min along the time axis, indicates a dramatic increase in PCA to 104.7 ± 3.6% ($P$ approximately 0.05). After this response, the PCA approached the baseline for the remaining perifusion period (Figure 4A). When bumetanide was added to EMP (40 mg $l^{-1}$), the acute response seen for EMP alone appeared unchanged during the first minute of perifusion (Figure 4B, see PCA at 12 min). In contrast to the curve with EMP alone, however, the PCA then rapidly approached the baseline during a 4-min period and the EMP effect (Figure 2C) was completely abolished. This is demonstrated by a background subtraction, in which the bumetanide curve was subtracted from the bumetanide/EMP curve (Figure 4C).

In Figure 5 furosemide (100 μmol $l^{-1}$) is shown to exhibit substantial effects on the PCA of glioma cells; the PCA increased rapidly to a maximum of 107.6 ± 1.6% ($P < 0.001$, $n = 5$) at 14 min of furosemide perifusion (Figure 5A) but then completely returned to the baseline with a negative slope value of –0.79 ± 0.16% $s^{-1}$ ($P < 0.001$) during the 14–24 min furosemide perifusion period. When furosemide (100 μmol $l^{-1}$) was added to the EMP (40 mg $l^{-1}$), the acute response seen for EMP alone seemed to be retarded in its increase, with a maximum at 13 min perifusion of 105.4 ± 1.8% ($P < 0.05$, $n = 5$, Figure 5B). This was of about the same magnitude as the acute response for EMP; however, note also that the PCA change clearly mimicked that for furosemide alone (Figure 2C). During the chosen time period of 14–24 min test perifusion, the significant slope observed for furosemide alone disappeared (Figure 5A). In addition, by the end of the 30-min test perifusion the PCA was 107.1 ± 2.0% ($P < 0.05$), and thus almost identical to the results for EMP alone (107.9 ± 2.2%). This is further strengthened by subtracting the curve for furosemide alone from the EMP–furosemide response (Figure 5B) (Figure 5A) with a resulting curve that shows a long-term EMP effect, whereas the acute EMP response was completely abolished (Figure 5C).

### DISCUSSION

In this study we show that the size of cultured glioma cells varies constantly during the cytotoxic action of EMP. Because of the rapid dynamics of these changes, we have focused on the possible role of ion channels in these volume-regulating mechanisms. This study was done with the use of a new computerized microscopic technique, which provides the ability to follow early alterations in cell size and shape of cells perifused with anticancer drugs.

EMP is a complex between oestradiol-17β and the alkylating agent nor-nitrogen mustard, and is widely used in the treatment of advanced prostatic cancer (Jönsson et al, 1977; Madajewics et al, 1980). We have recently shown that EMP is specifically metabolized by glioma cell in vitro (von Schoultz et al, 1990) and in vivo in rats (Bergenheim et al, 1993) and humans (Bergenheim et al, 1994). EMP and its main metabolite have demonstrated a marked cytotoxic effect on the cultured human malignant glioma cell line (MG-251) (von Schoultz et al, 1988), the cell line used in this study. The mechanisms of the cytotoxic action of EMP are still not completely understood. Earlier studies have suggested microtubules as the main target for the cytotoxic effects of EMP (Hartley-Asp, 1984; Wallin et al, 1985; Bjermer et al, 1988). Although EMP contains a highly active alkylating agent, it has been claimed that its cytotoxic effect is mediated through non-DNA targets (Tew et al, 1983). However, it has also been demonstrated that EMP cytotoxicity may involve a direct interaction with DNA and/or cell membrane components (Henriksson et al, 1990; von Schoultz et al, 1991). We have also shown that estramustine induces early DNA fragmentation, suggestive of an apoptotic cell death, in tumour tissue but not in brain tissue from the same animals (Vallbo et al, 1995). Moreover, EMP affects microtubule integrity and displays cytotoxic action in glioma cells but not in astrocytes (Yoshida et al, 1994). It is possible that the cell death induced by EMP may primarily be a membrane/cytoskeleton-triggered apoptotic cell death rather than a direct chemical interaction with the DNA.

Evidence that EMP cytotoxicity also involves a direct cell membrane damage was suggested in a previous study by (1) rubidium ($^{86}Rb$) accumulation assay, (2) scanning electron microscopy and (3) a new light microscopic technique of cell microperifusion (Engström et al, 1991). In addition, $^{86}Rb$ leakage was found to be a very early event following EMP exposure ($^{86}Rb$ is a commonly used tracer for potassium ion flux). In earlier studies, we had observed effects on $^{86}Rb$ fluxes following long-term EMP treatment in glioma cells (von Schoultz et al, 1991) and in transformed fibroblasts (Henriksson et al, 1990). Membrane damage might be related to previous observations that EMP, like other substances such as diamide and *tert*-butylhydroperoxide, is capable of generating free oxygen radicals. The direct involvement of free oxygen radicals in EMP toxicity has been shown in both a cell-free system (Grankvist et al, 1988) and in studies on different cell cultures (Henriksson et al, 1990).

Glioma cells demonstrate a consistent morphological alteration when exposed to EMP, starting at a concentration of 20 mg $l^{-1}$. EMP caused an obvious dose–response pattern in terms of increase in cell size without corresponding changes in shape factor. The cytotoxicity induced by EMP could be due to its microtubule depolymerization properties. This could be caused by interaction with tubulin and/or with microtubule-associated proteins (MPAs). Previous investigations have shown that high concentrations of EMP can inhibit microtubule polymerization in vitro by

© Cancer Research Campaign 1997

binding to MPAs. With this new computerized system no membrane blebs were detected as was the case in our previous report (Engström et al, 1991). This could be because of the difference in microscopy; phase contrast was used here rather than previous bright-field illumination and, further, the contour tracing was done interactively and not digitally (Engström et al, 1991). This discrepancy could also explain the lack of changes in shape factor observed with the previous technique.

The EMP-induced increase in cell volume indicates a membrane leakage, also shown by a decreased $^{86}$Rb influx with a net flow of ions and water into the cell (Engström et al, 1990). This ion and water leakage may either be of non-specific nature or occur by different ion channels (Hoffman and Simonsen, 1989). Perifusion of the glioma cells with ion-channel blockers ouabain, bumetanide or furosemide concomitant with EMP appeared to block the increase in cell volume induced by EMP. The actin cytoskeleton may be involved in detection of cell volume and/or transduction of a volume signal to volume-sensitive ion membrane transporters (Haas, 1994). It is thus possible that blockage of $Na^+$, $K^+$-ATPase and/or $Na^+$, $K^+$, $Cl^-$ co-transport activation by EMP could prevent morphological changes, DNA fragmentation and cytotoxicity of the drug. At least three different potassium flux pathways seem to exist in the tumour cell line used (malignant glioma U251 MG) i.e. $Na^+$, $K^+$-ATPase, $Na^+$, $K^+$, $Cl^-$ co-transport system and K channels with high conductance. The glioma cell line only displays moderate $Na^+$, $K^+$, $Cl^-$ co-transport activity, so it is perhaps not surprising that we did not find any reduction of the cytotoxic effect of EMP with furosemide and bumetanide in this cell line (Sandström et al, 1994).

In conclusion, this study shows that inhibitors of anion transmembrane transport completely block the EMP-induced cell volume increase. The interrelation between plasma membrane ion transport and cell volume changes by EMP, and the possible linkage to microtubule modulation and apoptosis-related DNA fragmentation are interesting observations that may help in solving the mechanisms of EMP cytotoxicity. The microperifusion technique for detecting and evaluating rapid cell volume changes in vitro could also serve as a valuable tool in predicting cytotoxicity during chemotherapy in clinical practice.

### ACKNOWLEDGEMENTS

This investigation was supported by grants from the Swedish Cancer Society and the Lion's Cancer Research Foundation, Umeå, and by the Lundberg's Research Foundation, Gothenburg, Sweden.

### REFERENCES

Bergenheim AT, Elfverson J, Gunnarsson P-O, Edman K, Hartman M and Henriksson R (1994) Cytotoxic effect and uptake of estramustine in a rat glioma model. *Int J Oncol* **5**: 293–299

Bergenheim AT, Gunnarsson P-O, Edman K, Von Schoultz E, Hariz MI and Henriksson R (1993) Uptake and retention of estramustine and the presence of estramustine binding protein in malignant brain tumours in humans. *Br J Cancer* **67**: 358–361

Bjermer L, Von Schoultz E, Norberg B and Henriksson R (1988) Estramustine inhibits monocyte phagocytosis. *Prostate* **13**: 49–55

Chapman JD, Peters LJ and Withers HR (eds) (1989) *Prediction of Tumor Treatment Response*. Pergamon: New York

Deweer P (1985) Cellular sodium–potassium transport. In *The Kidney, Physiology and Pathophysiology*. Seldin DW, Giebisch G (eds), pp 31–48. Raven Press: New York

Engström KG, Grankvist K and Henriksson R (1991) Early morphological detection of estramustine cytotoxicity measured as alteration in cell size and shape by a new technique of microperifusion. *Eur J Cancer* **27**: 1288–1295

Engström KG and Sävendahl L (1995) Cell volume and shape oscillations in rat type-II somatotrophs at hypotonic conditions. *Cytometry* **20**: 7–13

Grankvist K, Von Schoultz E and Henriksson R (1988) New aspects on the cytotoxicity of estramustine-involvement of free-oxygen radicals. *Int J Exp Clin Chemother* **1**: 37–42

Haas M (1994) The Na–K–Cl cotransporters. *Am J Physiol* **267**: C869–885

Hartley-Asp B (1984) Estramustine-induced mitotic arrest in two human prostatic carcinoma cell lines DU 145 and PC-3. *Prostate* **5**: 93–10

Henriksson R, Bjermer L, Von Schoultz E and Grankvist K (1990) The effect of estramustine on microtubule is different from the direct action via oxygen radicals on DNA and cell membrane. *Anticancer Res* **10**: 303–310

Hoffman EK and Simonsen LO (1989) Membrane mechanisms in volume and pH regulation in vertebrate cells. *Physiol Rev* **69**: 315–382

Jönsson G, Högberg B and Nilsson T (1977) Treatment of advanced prostatic carcinoma with estramustine phosphate (Estracyt®). *Scand J Urol Nephrol* **11**: 231–238

Madajewics S, Catane R, Mittelman A, Wajsman Z and Murphy GP (1980) Chemotherapy of advanced hormonally resistant prostatic carcinoma. *Oncology* **37**: 53–56

Sandström PE, Jonsson Ö, Grankvist K and Henriksson R (1994) Identification of potassium flux pathways and their role in the cytotoxicity of estramustine in human malignant glioma, prostatic carcinoma and pulmonary carcinoma cell lines. *Eur J Cancer* **30A**: 1822–1826

Tew KD, Eriksson LC, White G, Wang A, Shein PS and Hartley-Asp B (1983) Cytotoxicity of a steroid-nitrogen mustard derivative through non-DNA targets. *Mol Pharmacol* **24**: 324–328

Von Schoultz E, Grankvist K, Gustafsson H and Henriksson R (1991) Effects of estramustine on DNA and cell membrane in malignant glioma cells. *Acta Oncol* **30**: 719–723

Von Schoultz E, Gunnarsson PO and Henriksson R (1990) Uptake, metabolism and antiproliferative effect of estramustine phosphate in human glioma cell lines. *Anticancer Res* **9**: 1713–1716

Von Schoultz E, Lundblad D, Bergh J, Grankvist K and Henriksson R (1988) Estramustine binding protein and anti-proliferative effects of estramustine in human glioma cell lines. *Br J Cancer* **58**: 326–329

Vallbo C, Bergenheim AT, Bergh A, Grankvist K and Henriksson R (1995) DNA fragmentation induced by the antimitotic drug estramustine in malignant rat glioma but not in normal brain – suggesting apoptotic cell death. *Br J Cancer* **71**: 717–720

Wallin M, Deinum J and Friden B (1985) Interaction of estramustine phosphate with microtubuli-associated proteins. *FEBS Lett* **179**: 289–293

Yoshida D, Cornell-Bell A and Piepmier JM (1994) Selective antimitotic effects of estramustine correlate with its antimicrotubule properties on glioblastoma and astrocytes. *Neurosurgery* **34**: 863–867

*© Cancer Research Campaign 1997*